# Feed Space Allowance and Perch Design Criteria for Broiler Breeders Determined by Biometric Data

**DOI:** 10.3390/vetsci9070350

**Published:** 2022-07-11

**Authors:** Angela Gabriela Brandes, Birgit Spindler, Mona Franziska Giersberg, Nicole Kemper

**Affiliations:** 1 Institute for Animal Hygiene, Animal Welfare and Farm Animal Behaviour, University of Veterinary Medicine Hannover, Foundation, Bischofsholer Damm 15, 30173 Hanover, Germany; angela_brandes@hotmail.de (A.G.B.); nicole.kemper@tiho-hannover.de (N.K.); 2 Animals in Science and Society, Department Population Health Sciences, Faculty of Veterinary Medicine, Utrecht University, Yalelaan 2, 3584 CM Utrecht, The Netherlands; m.f.giersberg@uu.nl

**Keywords:** broiler breeder feet, body width, broiler weight, perching, enrichment

## Abstract

**Simple Summary:**

This investigation obtained data of broiler breeders’ body measurements to develop adjusted barn equipment, in order to realize high welfare standards and legal requirements. The broiler breeders had an average body weight of 2791.80 g (female) and 3615.88 g (male), and female broiler breeders had a body width of 20.63 cm, while it was 21.94 cm for the males. Accordingly, a trough side and perch length of 21.00 cm (female) or 22.00 cm (male), respectively, must be provided to ensure that all broiler breeders have equal access to feed and to perches. The feet showed an average length of 10.14 cm (female) and 12.05 cm (male). These measurements can be used for perch design.

**Abstract:**

The equipment used in broiler breeder houses is an important factor in allowing the expression of the various behaviours of the animals, and thus realizing high welfare standards. Presently, detailed requirements for the equipment in broiler breeder houses are not specified in Germany, especially feed space and perch design allowance. One reason is that basic biometric data on broiler breeders are lacking. To close this gap, a pilot study was conducted, and birds’ width, weight, and feet were measured. Broiler breeders at 22 weeks of age (50 female and 17 male) were weighed and photographed digitally, and their body widths were calculated from the photographs. Female broiler breeders weighed 2791.80 ± 334.99 g on average and showed a body width of 20.63 ± 1.88 cm. For males, a mean of 3615.88 ± 432.46 g was measured with a body width of 21.94 ± 2.32 cm. Our examinations revealed that a trough side length of 21.00 cm per hen and 22.00 cm per cock must be provided to ensure that all broiler breeders have equal access to feed. The same dimensions should be planned as the perch length for each animal. Measurements of broiler breeders’ feet (506 female and 150 male Cobb 500) were performed at 60 weeks of age. The length of each toe and the width and length of the foot pad of both sexes were photographed and measured digitally. Female broiler breeders’ feet showed an average length of 10.14 cm, and male birds showed a length of 12.05 cm. Based on recommendations for the perch design for laying hens, round and oval perches for broiler breeders should have a circumference of at least 11.30 cm (female) or 13.40 cm (male). For angular perches, the upper contact area should have a width of 2.80 cm (female) or 3.40 cm (male). The obtained biometric data could be a useful basis for the development of legal requirements for broiler breeders.

## 1. Introduction

Biometric data on animals offer the potential to design adequate housing environments and equipment for farm animals. This is decisively in order to provide farm animals with an animal-friendly husbandry environment that is adapted to the animal. In general, international and national legislation (e.g., Council Directive 1999/74/EC [1], TierSchG [2]) defines minimum standards for the protection of laying hens and requires that all animals kept for farming purposes be provided with appropriate space to meet their physiological and ethological needs. In addition to the space available, sufficient space at the feeding area and on perches are essential requirements for appropriate housing, especially for poultry. 

Apart from offering suitable perches, adequate space at the feeder is crucial for each broiler breeder. It is common practice to feed these birds restrictively in order to avoid extensive weight gain and the associated health impairments [3]. The degree of feed restriction relative to feeding ad libitum differs with age [3]. Nevertheless, feed restriction has a negative effect on welfare by leading to permanent hunger [4]. Usually, broiler breeders only have access to feed once a day for a limited time. Therefore, it is particularly important in restrictive feeding that all broiler breeders have access to feed at the same time. 

In general, separate-sex feeding systems to control the feed intake of males are widely used [5]. Management recommendations of breeding companies indicate a side length of the feeder of 15.00 cm for females and 20.00 cm for males when using chain feeding [5,6]. This calculation is based on the average width of full-grown breeder birds (12 to 15.00 cm at the widest part of a full-grown broiler breeder hen).

With regard to perches, in accordance with the Council of Europe [7], it is required for broiler breeders that every bird has the possibility to rest on a perch at night and that foot damage is avoided. The demand for perches is also supported by ethological studies. It has been clearly shown that perches of different height and design are used by broiler breeders [8,9]. Only in Switzerland [10] and Lower Saxony in Germany [11] are broiler breeder barns regularly equipped with perches to avoid foot damage and ensure animal welfare. In Switzerland, animal welfare regulations require seating accommodations at different heights according to the age and behaviour of broiler breeders [10]. The animal welfare control manual for Switzerland [12] details wooden, metal, or plastic perches, with at least two heights, a clear height of 50.00 cm over the perches, and a perch length of 14.00 cm when broiler breeder hens start laying. 

The federal state of Lower Saxony, in which a great share of Germany’s poultry farms are located, has also formulated minimum requirements for broiler breeder keeping [11]. This enactment requires all breeders to have access to an elevated place such as a perch or other plateau to rest during the dark phase. Perches must be gripped, their surfaces have to provide a secure hold, and their length has to offer 10.00 cm of space for every animal. 

For broiler breeders, exact perch design has not received much attention so far. In Germany, specific recommendations for laying hens are listed in the implementation notes of TierSchNutztV [13], which is a German Animal Welfare Ordinance. According to this, a physiological resting position requires that the toes are able to grip around the perch and find support. Furthermore, the foot pad should lie fully on the perch, which should not be slippery and should lack sharp splintering material. 

However, the recommendations for feeding space, perch space, and design are based on practical experience rather than on direct measurements of broiler breeders. To improve welfare, Riber et al. postulated that more research into effective environmental enrichment is needed [14]. Perching suggests the fulfilment of a behavioural need and could enhance animal welfare by assuring high production [9]. Investigations are necessary to collect basic data on broiler breeders and to provide adequate requirements. To meet these requirements, a collection of biometric data could provide assistance. For example, biometric measurements from pullets [15,16] have been used to define minimum space allowances and perch width during rearing when working on recommendations in Germany, as well as to provide useful information on the minimum space requirements and the design of housing equipment that have already been defined [17]. Thus, this pilot study was done to collect basic data on female and male broiler breeders’ body widths and feet measurements to create animal-based data for adapted equipment and requirements for broiler breeder houses.

## 2. Materials and Methods

### 2.1. Digital Measurement of Body Widths

The body widths of 50 female and 17 male broiler breeders (Cobb 500; Cobb Vantress Europe Ltd., Colchester, UK) with an age of 22 weeks were measured using the method described by Giersberg et al. [16] and Briese and Spindler [17]. From each broiler breeder, a frontal digital photo was taken under controlled conditions, and the body widths were calculated by a Python application (disto.py, 2009 Andreas Briese, Bri C Veterinärinstitut, Sarstedt, Germany).

The broiler breeders were housed in a commercial production farm in Germany with a flock size of about 5900 animals. The broiler breeders were floor housed with a littered scratching area and a raised slatted area next to the nests. The raised slatted area was equipped with perches and drinking lines. For the digital measurements of body widths, randomly selected broiler breeders were first weighed without prior selection (Digital balance Exceleron Acculab, Sartorius, Göttingen, Germany), and then photographed from a front view (Canon EOS 600D, Krefeld, Germany). The photos were obtained in the home barn to minimize the birds’ stress level. 

The birds were placed on a wooden perch (50.00 cm length × 110 cm height) with two black calibration marks that were 25.00 cm apart. After a short period of acclimatisation, the birds were photographed from a distance of 180.00 cm and a camera height of 110.00 cm in a standing position on the perch with legs stretched and wings folded to the body. Subsequently, the photographs were transferred to a personal computer and analysed using software. This calculation was based on counted pixels of the known width of the 25.00 cm reference standard and the known number of pixels of the connecting line between broiler breeder’s wings, and the body width of the birds (in cm) was found automatically according to the rule of proportion.

A trained observer marked the left and right outer contours of the broiler breeder’s wing at the level of the carpal joint (= body width) on each digital photo, as well as the horizontal distance between the two black calibration marks on the perch (= reference standard). Therefore, the software counted the horizontal pixels between these two calibration marks with the known width, as well as between the broiler breeder wings. Based on the pixel count, the known width of the reference standard (25 cm), and the known number of pixels of the connecting line between the marker points, the disto.py program calculated the body width of the broiler breeder (in cm) automatically according to the rule of proportion. All measurements were performed by one observer and were repeated three times per photo. The mean values were used for further analysis. A second trained observer verified the calculations for 20 birds (10 females and 10 males).

### 2.2. Digital Measurements of the Feet

The study examined 656 feet (506 from females and 150 from males) of broiler breeders to measure the size of the toes and food pads. The feet of male and female broiler breeders (Cobb 500, Cobb Vantress Europe Ltd., Colchester, UK) were sampled in a German slaughterhouse from a flock at the end of the production period with an age of 60 weeks. The feet of the separated slaughtered sexes were collected randomly without previous assessment from a running slaughter line.

To measure the basic biometric data of broiler breeder feet, the bottoms of the feet were photographed from the ventral bottom side with a digital camera (Canon EOS 600D, Krefeld, Germany) through a glass plate. The glass plate was installed on the top of a wooden photo box (70 cm × 40 cm × 30 cm, height × length × width). The digital camera was installed on the bottom of the box, and a remote release was activated with a cable release outside the box. The foot was placed on the top of the glass plate along with a measuring tape that was fixed on the glass plate for calibration marks. The feet were then photographed from the underside.

**Figure 1:**
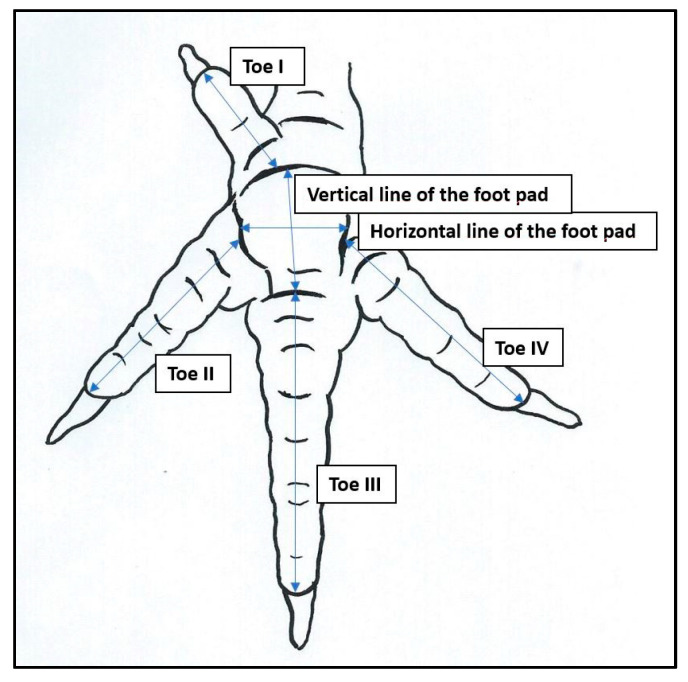
Measurements of the length of toe I, toe II, toe III, and toe IV, and the width and length of the foot pad (blue arrows) (illustration: A. Brandes).

Based on the digital photos, a digital evaluation was conducted using GNU Image Manipulation Program (GIMP) 2.8 (Mattis P. and Kimball S., GIMP 2.8, Berkeley, CA, USA, https://www.gimp.org/, accessed on 7 July 2022) [17]. The length and width were determined by counting the number of pixels of the different lengths and widths of the toes and foot pads and of the measuring tape with known distance. Each toe (toe I, toe II, toe III, and toe IV) was measured in the middle from the top to the foot pad without the claws. The foot pad was measured vertically and horizontally in the middle (Figure 1). The length of toe I, the foot pad, and toe III were added together to calculate the maximum foot length that can encompass a perch. 

All photographs and digital measurements were performed by one observer and were repeated three times per photo. The mean values were used for further analysis. A second observer verified the calculations for 20 feet. This separate dataset was evaluated by two experienced observers (a research scientist and veterinarian). Both observers were experienced in using the program.

### 2.3. Statistical Analyses

Descriptive statistics were calculated using Microsoft Office Excel 2007 (Redmond, Washington, DC, USA), and all other statistical analyses were performed using the software SPSS Statistics (version 27, IBM, New York, NY, USA). Observer reliability was calculated using the intraclass correlation coefficient and an absolute agreement definition in an evaluation that was carried out according to Cicchetti [18] (<0.4 = poor; 0.40–0.59 = moderate; 0.6–0.74 = good; ≥0.75 = almost perfect). Besides the performance of descriptive statistics, data were assessed for a normal distribution by creating histograms including a Gaussian distribution curve. Significant differences were identified between both sexes in terms of body weight, body width, toe length, and length and width of the foot pad by performing a t test for two independent groups (for body weight, toe length, foot pad length, and width) and a Mann–Whitney U test (for body width). The Levene test (in the form of an F-test) was always carried out in advance. Differences between the tested parameters were found to be significant if *p*-values were < 0.05.

## 3. Results

### 3.1. Observer Reliability

Calculations of observer reliability indicated ‘moderate’ and ‘almost perfect’ agreement between the observations. Values of the intraclass correlation coefficients for the different measurements can be found in Table 1.

### 3.2. Measurements of Broiler Breeder Body Width

**Figure 2:**
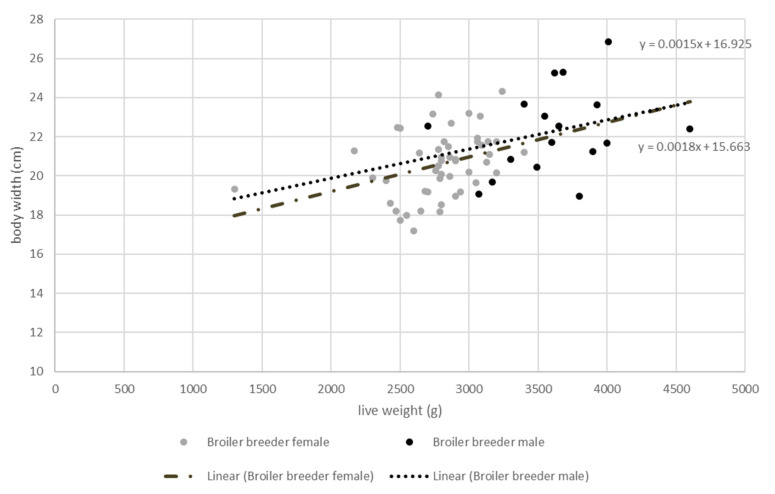
Body width (mm) of female (*n* = 50) and male (*n* = 17) 22-week-old broiler breeders (Ross 308) depending on their live weight (g).

The measurements of female broiler breeders (*n* = 50) revealed a mean live weight of 2791.80 ± 334.99 g and a mean body width of 20.623 ± 1.88 cm. The male broiler breeders (*n* = 17) had a mean weight of 3615.88 ± 432.46 g and were 29.52% heavier (*p* < 0.05) than the females, while their mean body width was 21.94 ± 2.32 cm, which was 6.37% wider (*p* < 0.05) (Figure 2). As expected, the body width and live weight had a positive correlation, as shown in Figure 2. Heavier animals had wider bodies, and vice versa.

### 3.3. Overall Measurements of Broiler Breeder Feet

In total, 656 feet of broiler breeders were measured, and the results are presented in Table 2. The length of broiler breeder toes varied between 2.23 ± 0.32 cm (female, toe I) and 6.91 ± 0.42 cm (male, toe III). In general, female broiler breeder feet were significantly shorter (*p* < 0.05) than male broiler breeder feet (toe I: 2.23 ± 0.32 cm vs. 2.46 ± 0.31cm; toe II: 3.87 ± 0.29 cm vs. 4.9 ± 0.35 cm; toe III: 5.65 ± 0.39 cm vs. 6.91 ± 0.42 cm; toe IV: 4.27 ± 0.31cm vs. 5.53 ± 0.35 cm; foot pad length: 2.26 ± 0.30 cm vs. 2.68 ± 0.37 cm; foot pad width: 2.20 ± 0.32 cm vs. 2.38 ± 0.27 cm). The mean toe lengths were shorter in females than males, with differences of 0.23 cm for toe I, 1.03 cm for toe II, and 1.26 cm for toes III and IV. 

**Figure 3:**
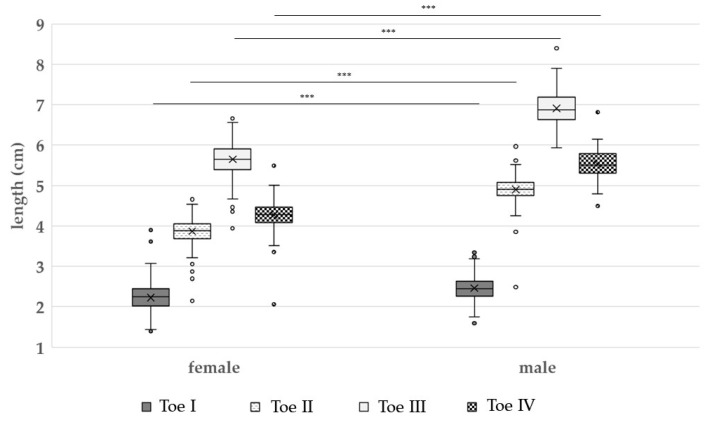
The lengths (cm) of toe I, II, III, and IV among female and male broiler breeders (*n* = 656 feet in total). Results are presented as boxplots (data range, median, lower quartile and upper quartile; outliers are included in the graph as dots and means as crosses). Significant effects between toe length of females and males are apparent in toe I, II, and III. *** = *p* < 0.05.

**Figure 4:**
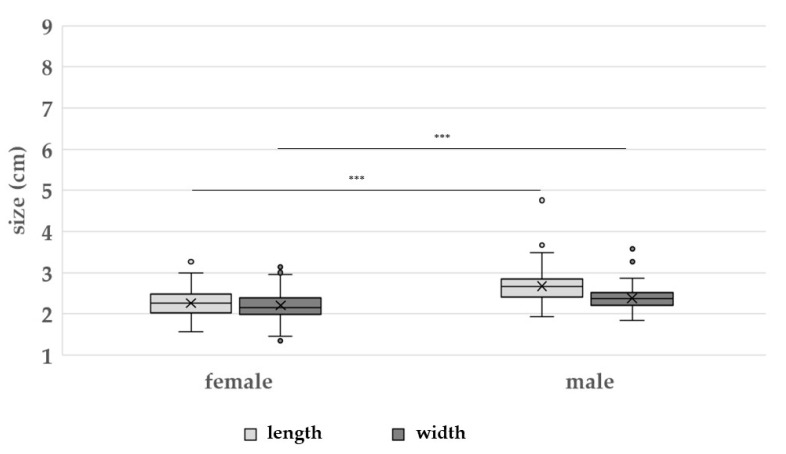
The lengths and widths (cm) of female and male broiler breeder foot pads (*n* = 656). Results are presented as boxplots (data range, median, lower quartile and upper quartile; outliers are included in the graph as dots and means as crosses). *** = *p* < 0.05.

On average, toe I was the shortest of all toes with a mean length of 2.23 ± 0.32 cm for female feet and 2.46 ± 0.31 cm for male feet (Figure 3). In contrast, toe III was the longest, with a mean of 5.65 ± 0.39 cm for female feet and 6.91 ± 0.42 cm for male feet. On average, the foot pad of the females showed a mean length of 2.26 ± 0.30 cm and a mean width of 2.20 ± 0.32 cm (Table 2). Compared to their longer toes (*p* < 0.05), the mean lengths and widths of males’ foot pads were also significantly larger (*p* < 0.05) than the female ones (Figure 4).

### 3.4. Deduction of the Foot Size for Perch Design

To calculate the total foot length that can encompass a perch, the length of toe I, the foot pad, and toe III were added together (Table 2). The maximum total foot length was 18.84% higher for male animals than females (*p* < 0.05).

## 4. Discussion

This basic study examined biometric data of broiler breeders’ body width and feet, and provides important data for animal-specific equipment in broiler breeder houses. As of 2010, the European Food Safety Authority (EFSA) [19] has required animal-specific equipment. Broiler breeder-specific data allows for the improvement of existing housing equipment, especially in feeder space and perch design, and can be used to enhance animal welfare legislation. 

The methods used here have already been used by scientists to collect biometric data on poultry [16,17]. Specifically, Briese et al. [17] collected width measurements of laying hens of different genetics. Giersberg et al. [16] conducted a survey of pullets during growth and defined minimum perch widths and feeder space allowances. Their results provide valuable data for the future design of perches for broiler breeders.

Our data on broiler breeder width were only collected once at the age of 22 weeks, so no definitive statements can be made about older animals. Nevertheless, the data provide valuable information on biometric data of broiler breeders. However, Briese and Spindler [17] and Giersberg et al. [16] found no effects of age or body position on the linear space in adult laying hens, which might also be applicable for broiler breeders. Our results show that sex has an effect on the measured body width: the males were wider than the females (21.94 ± 2.32 cm vs. 20.63 ± 1.88 cm at the same age, respectively). The larger dimensions of male animals were identical to planimetric measurements for the animal space of male and female broiler breeders [15].

Laying hens [20,21] and broiler breeders [12] show pronounced feeding synchronicity. Therefore, broiler breeders should provide adequate feeding space to feed at the same time when using a restriction-feeding program [22]. It is known that the incidence of aggression in a herd increases when poultry have to compete for limited feeder space [23]. According to their body width, the minimum trough lengths for linear track feeders should be 22.00 cm per bird for male and 21.00 cm for female broiler breeders. The recommended side lengths of commercial breeding companies are 15.00 cm for females and 20.00 cm for males when using chain feeding [12], which results in only about 70% of the females and 90% of the males being able to feed at the same time. 

The measurements of the feet of the broiler breeders provide valuable data for the design of perches. The biometric data go beyond the general requirements for the design of perches. Perches should maintain health and hygiene, and avoid injuries and impurities from excrements. Furthermore, they should have no sharp edges, unevenness, or materials that can hurt animals [3]. 

The measured body width data specific to broiler breeders indicate that previous housing equipment should be modified, especially feeder space and perch length. In order to allow all broiler breeders the possibility of resting on a perch, a perch length of 21.00 to 22.00 cm is necessary. This is confirmed by ethological studies, which found an intensive use of perches by broiler breeders, with up to 4.86 ± 0.65 birds/m during the dark period [4]. This corresponds to a space requirement of 20.57 cm/animal. 

The measurements of the feet were performed on the basis of photos taken in advance and subsequent pixel-based calculation of the lengths of individual toes and foot pads. A comparable method has already been used to measure toe and foot lesions of turkeys [24]. These photos were taken using a camera system that was installed at the end of a slaughter line, where feet are already separated from the body. The method used in our study has the advantage of photographing the feet from below while pressed on a glass plate, meaning the toes and the foot pads are optimally positioned. The measurements were clear, verifiable, and accordingly, more objective.

Thus far, only Pickel et al. [25] has measured the total feet length of poultry. They measured the feet of laying hens (Lohmann Selected Leghorn and Lohmann Brown hens) from plantar digit I to digit III, but the exact method was not described. The total feet length of Lohmann Selected Leghorn hens was 8.7 cm (±0.08 cm) with a body weight of 1761.2 g (±39.50 g), and those of Lohmann Brown hens were 9.2 cm (±0.07 cm) and 2053.9 g (±32.40 g), respectively [25]. In comparison, the Cobb 500 broiler breeders in our study had greater total feet lengths of 10.14 cm (±0.45 cm) for females and 12.05 cm (±0.59 cm) for males. For this result, the lengths of toe I, the foot pad, and toe III were summed. Similar to Pickel et al. [25], we also measured the feet of the broiler breeders without the claws. The claws at the top of the toes have different sizes and lengthen the toes, but it is obvious that it is the foot bones with the muscles that carry and balance the weight of the animals on the perches. Thus, the data recorded for laying hens in comparison to that of Pickel et al. [25] make it clear that due to the different results of the foot lengths, the application of data from one type of poultry to another is not meaningful. Rather, biometric data must be collected individually for each type of poultry.

**Figure 5:**
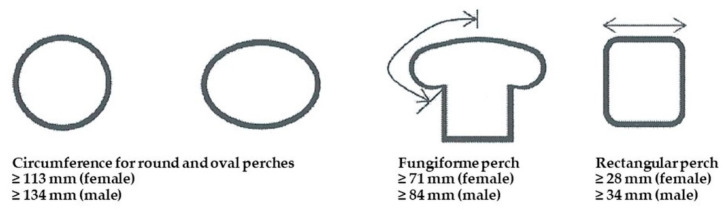
Recommended perch design for broiler breeders based on the results of this study (Illustration: A. Brandes; modified from the implementation instructions of TierSchNutzV).

The data can be used to design species-appropriate perches. Current requirements, as in the implementation instructions of TierSchNutzV [26], should ensure a safe foothold for laying hens, so it is necessary to allow grasping. The implementation instructions [26] require a circumference of at least 10.00 cm for oval or round perches (≈3.20-cm diameter for round perches). Angular perches must have rounded edges and a width of at least 2.50 cm. In the case of fungiformed perches for laying hens, the segment between the highest and the lowest points must be at least 6.30 cm long. This assumes a foot length of 9.00 cm, but the source of the measurement is unknown. However, this adopted foot length is comparable with measurements from Pickel et al. [25]. Transferring these requirements for laying hens to broiler breeders results in circumferences of at least 11.27 cm (females) and 13.39 cm (males) for oval and round perches (Figure 5). The lengths of tread surfaces of rectangular perches for broiler breeders must be calculated at 2.82 cm (females) and 3.35 cm (males), respectively. The segment between the highest and lowest point in fungiformed perches for broiler breeders should be at least 7.10 cm (females) and 8.44 cm (males) long (Figure 5). 

Apart from the form and surface area or diameter of perches, the height and material are important, too. Higher perches with a height of at least 5.50 cm seem to be preferred by broiler breeders [4]. With regard to the design of perches for laying hens, Pickel et al. found that perches with a soft surface may possibly reduce welfare problems related to the keel bone and foot pad [25]. Further balance movements should decrease with increasing perch diameter for laying hens, and appear less when using rubber perches compared to wood and steel [27]. Regarding landing behaviour, steel perches were found to be the least advantageous [28]. Moreover, the perch temperature can affect laying hens’ resting behaviour [29]. Wooden perches covered with rubber burden the foot pads in an unphysiological way [30].

## 5. Conclusions

In conclusion, our study provides useful basic biometric data on broiler breeders. Digitally exact measurements of the foot pad, length of the toes, and body width are needed to meet legal requirements. There are differences between laying hens and broiler breeders. Broiler breeders show the characteristic perching behaviour of *Gallus gallus*, so providing perches is essential for their welfare. The equipment used in broiler breeder houses should be adjusted to their body measurements because they are not comparable with other poultry. Optimally adjusted equipment supports animal health, welfare, and performance in production. Recommendations of ethological studies should be substantially validated.

## Figures and Tables

**Table 1:** Observer reliabilities for the measured body width, toe length, and food pad width and length (*n* = 20 birds and 20 feet).

Parameter	Interobserver Reliability OB 1 vs. OB 2	Intraobserver Reliability OB 1
Body width	0.847	0.932
Toe I	0.688	0.989
Toe II	0.756	0.942
Toe III	0.918	0.909
Toe IV	0.498	0.512
Foot pad length	0.702	0.880
Foot pad width	0.530	0.579

OB = Observer.

**Table 2:** Measurements (cm) at time of slaughter of toe I–IV and foot pads of female (*n* = 506 feet) and male (*n* = 150 feet) broiler breeders, as well as length and width and the foot length in total (toe I + foot pad length + toe III).

	Toe I		Toe II		Toe III		Toe IV		Foot Pad Length		Foot Pad Width		Foot Length in Total
Sex	Mean ± SD	Range	Mean ± SD	Range	Mean ± SD	Range	Mean ± SD	Range	Mean ± SD	Range	Mean ± SD	Range	Mean ± SD
female	2.23 ± 0.32	1.40–3.90	3.87 ± 0.29	2.15–4.66	5.65 ± 0.39	3.94–6.69	4.27 ± 0.31	2.06–5.49	2.26 ± 0.30	1.57–3.27	2.20 ± 0.32	1.35–3.20	10.14 ± 0.45
male	2.46 ± 0.31	1.59–3.34	4.90 ± 0.35	2.49–5.97	6.91 ± 0.42	5.93–8.40	5.53 ± 0.35	4.50–6.83	2.68 ± 0.37	1.94–4.76	2.38 ± 0.27	1.84–3.58	12.05 ± 0.59
*p*-value	*p* < 0.05		*p* < 0.05		*p* < 0.05		*p* < 0.05		*p* < 0.05		*p* < 0.05		*p* < 0.05

## Data Availability

The data presented in this study are available upon reasonable request from the corresponding author. The data are not publicly available due to privacy.
